# Influence of sonication on the phenolic content and antioxidant activity of *Terminalia catappa* L. leaves

**DOI:** 10.4103/0974-8490.75457

**Published:** 2010

**Authors:** H. V. Annegowda, L. N. Anwar, M. N. Mordi, S. Ramanathan, S. M. Mansor

**Affiliations:** *Centre for Drug Research, Universiti Sains Malaysia, 118 00 Penang, Malaysia*

**Keywords:** Antioxidants, DPPH, FRAP, *T. catappa*, Sonication, Vitamin C

## Abstract

**Aim:**

This study was designed to evaluate the phenolic content and antioxidant activity of ethanolic extracts from *T. catappa* leaves obtained by different intervals of sonication.

**Methods:**

Three commonly used methods were followed to evaluate phenolic content and four in vitro methods like 2,2-diphenyl-1-picrylhydrazyl (DPPH), 2,2-azinobis-(3-ethylbenzothiazoline-6-sulfonic acid) diammonium salt (ABTS) radical scavenging assay, ferric reducing antioxidant potency (FRAP), and total antioxidant capacity assays for measuring the antioxidant activities. Antioxidant values of these assays were expressed in terms of milligrams vitamin C equivalent (VCE) antioxidant activities.

**Results:**

This study showed that extract obtained with 40 minutes of sonication possessed significant (*P* < 0.05) polyphenolic contents compared to 20 and 60 minutes sonication and control (24 hour maceration). Moreover, sonication of *T. catappa* leaf above 40 minutes was found to be unsuitable for extracting out phenolic contents. Even the results of antioxidant assays showed that 40 minutes of the sonicated extract exhibited significant (*P* < 0.05) VCE values compared to extracts obtained at different intervals of sonication and control.

**Conclusions:**

In sonication extraction method 40 minutes is an ideal time to obtain extract enriched with high polyphenolic content with good antioxidant activity from *T. catappa* leaves.

## INTRODUCTION

Oxidative stress is involved in the pathophysiology of various chronic diseases, such as, cardiovascular disease, neuronal disease, cataracts, and several types of cancer, and antioxidants play an important role in the prevention of these diseases.[1] In this context, there is an increasing interest to search for natural antioxidants present in functional herbs, fruits, and vegetables. Phytoconstituents such as phenolics, carotenoids, anthocyanins, and tocopherol have been found to exert chemopreventive[2] and cardioprotective effects.[3]

*Terminalia catappa* L (*T. catappa* L) is a Combretaceous plant, which grows commonly in the tropical and subtropical countries. The leaf, bark, and fruit of this plant have long been used in folk medicine for antidiarrheic, antipyretic, and hemostatic purposes in India, Phillippines, Malaysia, and Indonesia.[4] The *T. catappa* leaf has been reported to possess hepatoprotective, anti-inflammatory, and anti-HIV reverse transcriptase activity.[5–7] The fallen leaves are boiled or brewed with water and used as a drink. Leaves of *T. catappa* contain a number of hydrolysable tannins such as punicalagin, punicalin, chebulagic acid, corialgin, geranin, terflavins A and B, tergallagin, gratin B, and triterpenoids, such as, ursolic acid and 2α, 3β, and 23-trihydroxyurs-12-en-28 oic acid.[6,8]

Extraction is an important step involved in the discovery of bioactive components from plant materials. Recent studies have shown that sonication-assisted extraction enhances the extraction efficiency by achieving a higher yield of plant secondary metabolites, at a reduced processing time, solvent consumption, and at a lower temperature.[9–11] All these features make sonication an attractive technique for the extraction of antioxidants from *T catappa* leaves. Vitamin C is commonly recognized as a major natural antioxidant and nutrient in our diet and is found to possess anti-carcinogenic activity.[12] Of late, more attention has been focused on the determination of the total antioxidant capacity of compounds using the vitamin C equivalent antioxidant capacity (VCEAC) assay.[13] The VCEAC assay is a very popular assay and the value is assigned by comparing the antioxidant capacity or radical scavenging ability of an antioxidant to that of vitamin C.

*T. catappa* leaves were found to possess good antioxidant activity, reducing power and inhibitors of peroxidation.[14,15] Thus far, classical solvent extraction, soxhlet extraction, and supercritical fluid extraction have been used for the extraction of *T. catappa*.[14,15] To our knowledge, to date, no investigation has been reported with regard to the comparison of phenolic content and antioxidant activity of *T. catappa* extracts, obtained by different intervals of sonication. Hence, the primary objective of our study was to evaluate the influence of sonication on the phenolic content and to provide bioactivity information of the ethanolic extract of *T catappa* leaves for the development and application of the resource, and our secondary objective was to develop the concept of VCEAC, to express the free radical scavenging ability and antioxidant activities of the sonicated ethanolic extract of *T catappa* leaves.

## MATERIALS AND METHODS

### Plant material

Fresh leaves of *Terminalia catappa* were collected from the campus of the Universiti Sains Malaysia (USM), Penang, Malaysia, in November. The plant was authentified by a botanist and the voucher specimen (No 11048) was deposited in the Herbarium, School of Biological Sciences, USM. The collected leaves were then washed with running water to remove the dirt, dried, and ground to obtain powder of 40 mesh size.

### Chemicals

2,2-diphenyl-1-picrylhydrazyl (DPPH), 2, 4, 6-tris(2-pyridyl)-1, 3, 5-triazine (TPTZ), 2,2-azinobis-(3-ethylbenzothiazoline-6-sulfonic acid) diammonium salt (ABTS), Folin–Ciocalteu reagent, potassium persulfate, vanillin, sodium carbonate, aluminium chloride hexahydrate, ferric chloride, sodium acetate, ascorbic acid, gallic acid, and catechin were purchased from Sigma Chemicals, Germany. All the other solvents used in this study were of analytical grade and purchased from Fisher Scientific Sdn. Bhd, Malaysia.

### Ultrasonic extraction

Twenty-five grams of leaf powder was mixed with 250 mL of ethanol (99.5%) separately in a beaker and covered with aluminum foil. This mixture was then sonicated at different intervals of 20, 40, and 60 minutes in the dark, at room temperature. A control sample was prepared without sonication (maceration for 24 hours). The ultrasonic treatment was performed using a Branson 5510 ultrasonic cleaner (42 kHz, 135 W; BRANSON ULTRASONIC CORPORATION, USA). The solvent surface in the beaker was kept at the level of the water in the ultrasonic bath, with the water being circulated and regulated at constant room temperature, to avoid a rise in water temperature caused by the ultrasonics. After the extraction, the sample residues were dissolved in a known quantity (100 mL) of ethanol and were repeatedly extracted until the extracts became clear. Then all the extracts were filtered and the filtrates obtained were then concentrated using a rotary evaporator, dried in a freeze-dryer, and kept at 4°C in air-tight containers until further analysis.

### Determination of total phenolic content, total flavonoids, and total tannins

The total phenolic content of the *T catappa* sonicated ethanolic (TCSE) extracts were determined using the Folin-Ciocalteu colorimetric method and the values were expressed in terms of milligram gallic acid equivalents (mg GAE) per gram of extract.[16] The total flavonoid content in the extracts was carried out using the Aluminium chloride colorimetric method and the values were expressed in terms of milligram catechin equivalents (mg CAE) per gram of extract.[17] However, the vanillin-HCl method with slight modifications was used to measure the total tannin concentrations in the extracts, and the values were expressed in terms of milligrams catechin equivalents (mg CAE) per gram of extract.[18]

### ABTS radical scavenging assay

The ABTS radical scavenging activity of the TCSE extract was determined according to the previously described procedure.[19] Fresh ABTS solution was prepared by adding 5 mL of a 4.9 mM potassium persulfate solution to 5 mL of a 14 mM ABTS solution and this solution was kept for 16 hours in the dark, at room temperature (25 ± 1 °C). This solution was diluted with methanol to yield an absorbance of 0.700 ± 0.02 at 734 nm and the same solution was used for the antioxidant assay. The final reaction mixture (1 mL) of standard and extracts comprised 950 µL of the ABTS solution and 50 µL of the sample. This reaction mixture was vortexed for 10 seconds, after six minutes, absorbance was recorded at 734 nm using a UV-visible spectrophotometer (Shimadzu UV-1800, Kyoto, Japan) and compared with the control ABTS solution. The vitamin C calibration curve was prepared by plotting the percentage inhibition of vitamin C at various concentrations (5 – 100 µg/mL). The percentage inhibition was calculated using the following formula:

Percentage inhibition of Vitamin C and extracts

$$=\;\left[\left(A_{0}\;-\;A_{1}/A_{0}\right)\;\times \;100\right]$$

where A_o_ was the absorbance of the control, and A_1_ was the absorbance of samples.

The results were expressed as the milligrams vitamin C equivalent radical scavenging (VCERS) ability per gram of extract.

### DPPH radical scavenging assay

The DPPH radical scavenging activity of the TCSE extracts were measured according to the method described by Brand-Williams *et al*.[20] Briefly, to 0.1 mL of crude extract (100 µg/mL) 2.9 mL of DPPH solution (100 µM in methanol) was added. Further, this solution was incubated in the dark for 30 minutes, at room temperature. The decrease in the absorbance (due to the proton-donating activity) was measured at 517 nm against a blank consisting of 0.1 mL of ethanol and 2.9 mL of DPPH solution. The vitamin C calibration curve was prepared by plotting the percentage inhibition of vitamin C at various concentrations (25 – 250 µg/mL). The percentage inhibition was calculated using the formula mentioned in the DPPH assay and the results were expressed as milligrams VCERS ability per gram of extract. All the measurements were done in triplicate.

### Ferric reducing antioxidant property

The ability of the TCSE extracts to reduce the ferric ions was measured according to the modified method described by Benzie and Strain.[21] Briefly, a known aliquot of the sample (200 µl) was added to 3 ml of a FRAP reagent (10 parts of 300 mM sodium acetate buffer at pH 3.6, one part of 10 mM TPTZ solution, and one part of 20 mM FeCl_3_·6H_2_ O solution), and the reaction mixture was incubated in a water bath (at 37°C). The increase in absorbance at 593 nm was measured after a 30 minute time interval. Ascorbic acid in various concentrations (5 – 100 µg/mL) was used for the preparation of the standard calibration curve. The FRAP values were expressed as milligrams vitamin C equivalent ferric reducing antioxidant potency (mg VCEFRAP) per gram of plant material.

### Total Antioxidant capacity

The method described by Prieto, P *et al*.[22] was adapted to measure the antioxidant capacity of the TCSE extracts. In brief, to a known aliquot of the sample solution (0.4 ml) taken in a vial, 4 ml of the reagent solution (0.6 M sulfuric acid, 28 mM sodium phosphate, and 4 mM ammonium molybdate) was added and incubated in a water bath at 95°C for 90 minutes. The blank solution contained 4 ml of reagent solution and 0.4 ml of methanol. After the samples were cooled to room temperature (25 ± 1°C), the absorbance was measured at 695 nm against a blank. The calibration curve was prepared by using a standard solution of ascorbic acid (5 – 100 µg/ml) and the antioxidant activity was expressed as milligrams vitamin C equivalent antioxidant capacity (VCEAC) per gram of extract.

### Statistical analyses

The results of the present study are presented as mean ± SD (n = 3). Analysis of variance was performed (one way ANOVA) and the significant differences between the mean values were determined by Tukey’s pairwise test, at a level of significance of *P* < 0.05. Statistical analyses were carried out using SPSS 17 (SPSS Inc. USA).

## RESULTS

In the present study, the *T catappa* leaf was extracted by sonication at a different interval of time using ethanol as the solvent. From the results of the extraction [Table 1], it was seen that the maximum extraction yield was obtained by 60 minutes of sonication in comparison with the control, 20- and 40-minute intervals of sonication. Moreover, as the sonication interval increased there was a corresponding increase in the extraction yield, but the extraction yield of 20 and 40 minutes of sonication was almost the same as that of the control.

**Table 1:** Effects of sonication treatments on percentage yield and phenolic contents in TCSE extra

Extracts	Yield (%)	Total phenolic^1^	Total flavonoids^2^	Total tannins^2^
Control	5	234.45 ± 0.62^c^	50.77 ± 1.83^a^	32.10 ± 0.85^a^
Sonicn 20 minutes	4.8	223.61 ± 0.73^a^	57.74 ± 1.67^b^	36.80 ± 0.80^b^
Sonicn 40 minutes	5.3	238.36 ± 1.12^d^	74.91 ± 2.48^d^	35.73 ± 1.02^b^
Sonicn 60 minutes	6.4	230.14 ± 0.53^b^	68.75 ± 1.53^c^	31.90 ± 0.50^a^

1 mg gallic acid equivalents / g sample

2 mg catechin equivalents / g sample

Sonication (Sonicn) and Control (24-hour maceration); Values with different alphabets in the same column were significantly different (*P* < 0.05) from each other. Values are the means of three replicates ± standard deviation (SD)

The results shown in Table 1 clearly indicate that as the sonication interval increases there is a corresponding increase in the total phenolic and flavonoid content in the TCSE extracts. Both these values are significant (*P* < 0.05) for the extract obtained with 40 minutes of sonication, as compared to all the other extracts, and the values range from 223.61 ± 0.73 to 238.36 ± 1.12 mg GAE/g extract and from 50.77 ± 1.83 to 74.91 ± 2.48 mg CAE/g extract for total phenols and total flavonoids, respectively. However, both 20 and 40 minutes of sonicated extracts possess almost an equal and significant amount (*P* < 0.05) of total tannins compared to the control and 60 minutes of sonication. The total tannin content varied from 31.90 ± 0.50 to 36.80 ± 0.80 mg CAE/g extract.

Results of the DPPH and ABTS assays are summarized in the Table 2. The free radical scavenging ability of TCSE extracts are expressed in terms of mg VCERS ability/g extract, which is more appropriate than expressing the values in terms of percentage inhibition or decrease in absorbance. These values are calculated using the vitamin C standard curve.[13] As displayed in Table 2, the VCERS ability for these extracts has a wide variability and ranges between 329.76 ± 9.73 to 388.01 ± 12.58 and 396.23 ± 9.71 to 433.10 ± 8.32 mg VCERS ability/g extract for the DPPH and ABTS assays, respectively. Extracts obtained after 40 minutes of sonication exhibit a significantly high (*P* < 0.05) vitamin C equivalent value compared to all the other extracts. Moreover, in the DPPH assay even 60 minutes of sonication shows significant (*P* < 0.05) VCERS compared to the control, whereas, in the case of the ABTS assay [Table 2], there is no significant vitamin C equivalent value that exists among the control, 20, and 60 minutes of sonication.

**Table 2:** Effects of sonication treatments on antioxidants in TCSE extracts

Extracts	ABTS assay^1^	DPPH assay^1^	FRAP assay^2^	Total antioxidant capacity assay^3^
Control	404.06 ± 6.82^a^	329.76 ± 9.73^a^	213.92 ± 0.56^bc^	329.64 ± 1.76^c^
Sonicn 20 minutes	397.16 ± 5.58^a^	336.61 ± 9.27^ab^	209.70 ± 1.01^a^	311.19 ± 1.63^a^
Sonicn 40 minutes	433.10 ± 8.32^b^	388.01 ± 12.58^c^	215.44 ± 0.67^c^	334.29 ± 1.88^d^
Sonicn 60 minutes	396.23 ± 9.71^a^	360.59 ± 9.02^b^	212.19 ± 0.72^b^	319.11 ± 1.17^b^

1 mg VCERS ability/g sample

2 mg VCEFRAP/g sample

3 mg VCEAC/g sample; Control (24-hour maceration)

Values with different alphabets in the same column were significantly different (*P* < 0.05) from each other. Values are the means of three replicates ± standard deviation (SD)

The results of the FRAP assay varied from 209.70 ± 1.01 to 215.44 ± 0.67 mg VCEFRAP/g extract [Table 2]. The extract with 40 minutes of sonication possessed a significant (*P* < 0.05) vitamin C equivalent value, enough to reduce the ferric ion to ferrous ion, compared to 20 and 60 minutes of sonication, but in comparison with the control, the value seemed to be not that significant (*P* > 0.05). However, in the total antioxidant capacity assay, the extract with 40 minutes of sonication exhibited significant(*P* < 0.05) VCEAC values compared to all the other extracts. The VCEAC values varied from 311.19 ± 1.63 to 334.29 ± 1.88 mg VCEAC/g extract; even in this assay the VCEAC values for 20 and 60 minutes of sonication were not significant (*P* > 0.05) in comparison with the control.

## DISCUSSION

Sonication-assisted extraction was found to be very simple, inexpensive, and faster, operated at a lower temperature, and was an efficient alternative to conventional extraction techniques.[23] Ethanol was found to be an ideal solvent in sonication-assisted extraction, to leach out bioactive compounds from dried plant material.[24] The cavitational effects were reduced as the temperature was increased, hence, in this study we carried out sonication at room temperature.[25] Forty minutes of sonication wasfound to enhance the extraction yield of the TCSE extract compared to the control (24 hours), which could be due to the cavitational effect that led to tissue disruption and a good penetration of the solvent into the tissue matrix.[26]

As is evident from Table 1, the extraction yield and the total phenolic and total flavonoids increased rapidly from 20 to 40 minutes of sonication and started decreasing as the sonication was prolonged. We suspect that it could be due to the decomposition of the bioactive constituents by prolonged sonication or due to the initial rinsing effect of sonication, which facilitated the release of most of the active constituents inside the cells to the solvent during the first 40 minutes. Furthermore, prolonged sonication led to a decreased diffusion area, diffusion rate, and increased diffusion distance, which could led to a decreased yield of total phenolic and flavonoid content at a prolonged interval of sonication. A similar finding was also reported by Lu *et al*.,[27] wherein, they mentioned that sonication above 40 minutes led to decreased yield of anthraquinone from *Rheum palmatum*. Hence, sonication for more than 40 minutes in ethanol appeared to be disadvantageous. However, sonication was found to be an ideal method to extract greater amounts of flavonoids compared to the control. The ideal time interval of sonication to extract the maximum amount of tannin content from *T catappa* leaves was found to be 20 minutes. Sonication for more than 20 minutes was found to be unsuitable, as there was not a great amount of tannin content extracted by increasing the time interval. It was also clear from the results that the total phenolic compounds were not related to the extraction yield, but depended on the interval of sonication.

The results of a single experiment will give only a reductive suggestion about the antioxidant properties of the sample evaluated and must be interpreted very cautiously. Therefore, an approach with multiple assays for antioxidant properties is appropriate.[28,29] In this respect, TCSE extracts were subjected to four different *in vitro* antioxidant assays such as ABTS, DPPH, FRAP, and total antioxidant capacity assays. DPPH and ABTS assays act by reduction of preformed radicals and determine the decrease in the absorbance, while the FRAP and total antioxidant capacity assays measure the increased absorbance of the formed ferrous ion and Mo(V) complex, respectively. Antioxidant activity measured in terms of mg VCEAC/g extract is more meaningful and descriptive, as the results provide a direct comparison of antioxidant activity of the sample with vitamin C.[13]

DPPH and ABTS assay methods are the most popular spectrophotometric methods for determination of the radical scavenging activity of herbs, food, and vegetables. These two stable radicals are easy to use and also have a high level of sensitivity, even when utilized for determination of the antioxidant activity of numerous pure compounds, vegetables, fruits, and tea extracts.[19,30–32] As is evident from the results of the ABTS and DPPH assays [Table 2], 40 minutes of sonicated extract is shown to exhibit significantly higher (*P* < 0.05) VCERS activity compared to the control, 20 and 60 minutes of sonication. This indicates that a higher amount of antioxidant constituents is extracted by 40 minutes of sonication. It is even evident from the results of the phenolic content determination that the extract obtained by 40 minutes of sonication exhibits significant amounts of phenolic, flavonoid, and tannin contents. Even though, VCERS values of these extracts are good, a weak correlation is observed among the VCERS values of DPPH, ABTS assays, and phenolic content. We suspect, the good VCERS activity of these extracts may be due to the synergism between the phenolic and non-phenolic compounds present in these extracts.

The FRAP assay is based on the reduction of the ferric tripyridyl triazine (Fe^3+^ -TPTZ) complex to its colored ferrous tripyridyl triazine (Fe^2+^ -TPTZ) form and the assay directly measures the amount of antioxidants or reductants in the sample.[21] VCEFRAP values of extracts are calculated using the vitamin C standard curve.[13,33] As seen in Table 2, the extracts obtained at different intervals of sonication exhibit various degrees of VCEFRAP values, in which 40 minutes of sonication exhibits a significant VCEFRAP value compared to the other extracts. A strong correlation is found between the total phenolic and VCEFRAP values (R^2^ = 0.9998), which ensures that phenolic compounds may be responsible for the antioxidant activity. The total antioxidant capacity method is based on the reduction of Mo(VI) to Mo(V) by the antioxidants in the samples, resulting in the formation of a green Mo(V) complex, with the absorption maximum at 695 nm.[22] A similar trend of the FRAP assay is also observed here, as the VCEAC value for the extract obtained by different intervals of sonication varies and exhibits a varied antioxidant value. Moreover, a strong correlation is also found for the total phenolic and mg VCEAC/g extract of total antioxidant capacity assay (R2 = 0.9788), which ensures involvement of polyphenols in the reduction of Mo(VI) to Mo(V). A similar finding is also reported by Jayaprakasha *et al*.,[34] wherein, grape seeds that are obtained with different solvents exhibit various degrees of antioxidant capacity.

## CONCLUSION

In the present study, we have investigated the influence of sonication on the phenolic content from *T. catappa* and evaluated the antioxidant activity using *in vitro* methods, including DPPH, ABTS, FRAP, and total antioxidant capacity assays. The results of this study indicated that 40 minutes of the sonication process will help in getting an extract enriched with significant polyphenolic content, which may serve as a source of natural antioxidant. Our study also ensured that prolonged sonication (more than 40 minutes) is not recommended for the extraction of antioxidant bioactive components, as it may be lead to the degradation of the active constituents. Moreover, in this study, the antioxidant activity of TCSE extracts were expressed in terms of milligrams VCE antioxidant activity per gram of extract, which was found to be an easy and ideal method to adapt to compare the activity of any extract with that of vitamin C. The results of our study also emphasized the use of at least two test systems, due to the difference between the methods investigated. Further experiments are underway to find the bioactive compounds responsible for the varied phenolic content and antioxidant results, and also to find the antioxidant activity in the physiological environment of the body.
